# A multivariate morphometric investigation to delineate stock structure of gangetic whiting, *Sillaginopsis panijus* (Teleostei: Sillaginidae)

**DOI:** 10.1186/s40064-016-2143-3

**Published:** 2016-04-26

**Authors:** Muhammad Abu Bakar Siddik, Md. Abu Hanif, Md. Reaz Chaklader, Ashfaqun Nahar, Ravi Fotedar

**Affiliations:** Department of Fisheries Biology and Genetics, Patuakhali Science and Technology University, Patuakhali, 8602 Bangladesh; Department of Marine Fisheries and Oceanography, Patuakhali Science and Technology University, Patuakhali, 8602 Bangladesh; Department of Environment and Agriculture, Curtin University, 1 Turner Avenue, Bentley, WA 6102 Australia

**Keywords:** Morphometric characters, Univariate ANOVA, Sustainable management, *Sillaginopsis panijus*

## Abstract

This study was conducted to delineate the stock structure of *Sillaginopsis paniijus* based on morphometric characters of the species. A total of 194 specimens were collected from the Meghna, Tentulia and Baleswar rivers located in the southern coastal zone of Bangladesh. Data were subjected to univariate ANOVA, multivariate ANOVA, discriminate function analysis (DFA), and principal component analysis. Mean variations of ten morphometric characters; HD, HBD, LBD, PsOL, ED, SnL, SPrDL, HAF, LSDB and LPB showed significant differences (*p* < 0.05) among 27 morphometric traits that were selected for the study. In DFA, the overall assignments of individuals into their correctly classified original groups were 71.1 and 70.6 % for male and female, respectively. A scatter plot of the first two discriminant functions was used to visually depict the discrimination among the populations. The results showed different stocks of *S. panijus* in the rivers of Baleswar, Tentulia and Meghna in southwest coast of Bangladesh.

## Background

Morphometric characters is considered one of the simplest, most cost-effective and most commonly used tools to identify and characterize fish stocks (Cadrin and Silva 2005; Chaklader et al. 2015; Siddik et al. 2016), in determining the structure of fish assemblages (Cheng et al. 2005) and to distinguish between fish populations (Cheng et al. 2005; Siddik et al. 2015). In the past, scientists assumed variation of morphometric characters was entirely genetic, but recent studies have proved its relation with environmental factors including physico-chemical parameters of water, habitat and substrate types (Cabral et al. 2003; Nahar et al. 2015; Sharker et al. 2015). Although genetic and physiological differences between stocks revealed by molecular markers is more trustworthy, morphometric variations are still considered an important tool in stock characterization and identification (Costa et al. 2003; Murta 2000). Morphometric characters can be used to quantify a trait having evolutionary significance by detecting changes in the shape (Chaklader et al. 2016a). Therefore, the morphological studies on fishes can potentially contribute to better management and conservation strategies for a population (Muchlisin et al. 2014) and lead to a better understanding of species evolution, ecology, behavioral traits and stock assessment (AnvariFar et al. 2011; Chaklader et al. 2016b).

Gangetic whiting, *Sillaginopsis panijus* is an inshore marine and estuarine fish species from the family Sillaginidae under the order of Perciformes. This species also called ‘Flathead Sillago’ due to its highly depressed head, very small eyes limited by the orbits and because its second spine among the ten spines of the first dorsal fin is highly modified (McKay 1992). The *S. panijus* is found along the south west coast, Gangetic delta, coast of Bangladesh (Hanif et al. 2015a) and some parts of India, Myanmar, Malaysia and infrequently in the Indonesian archipelago (Azim et al. 2012). This species is, however, more abundant in the southwest coastal rivers and estuaries of Bangladesh than in any other locations in its wide geographical distribution (Hanif et al. 2015b). It can grow up to a total length of 44 cm (Froese and Pauly 2007). The *S. panijus* has high demand in local markets but rarely in overseas markets, although juvenile species are occasionally traded as aquarium fish. Based on its high commercial value and socio-economic importance, a thorough study of the species was deemed essential to get a better understanding of its morphology, biology, habitats, ecology and also culture system of this species.

Recent investigation indicated that there are severe declining *S. panijus* populations all along the southern coast of Bangladesh due to overfishing, introduction of exotic species, pollution, damming and even global climate change consequences (Siddik et al. 2015). To conserve this species abundance in this vast area, the life history studies of this species should be given utmost priority. Therefore, the present study was considered as a first step towards exploring the stock structure of this species based on morphometric characters for its sustainable development and management across the southern coast of Bangladesh.

## Methods

### Ethics statement

The ethical issue is not required for the described study in Bangladesh. The field location is not privately owned or protected in any way, and the study did not involve endangered or protected species.

### Collection of samples

**Fig. 1:**
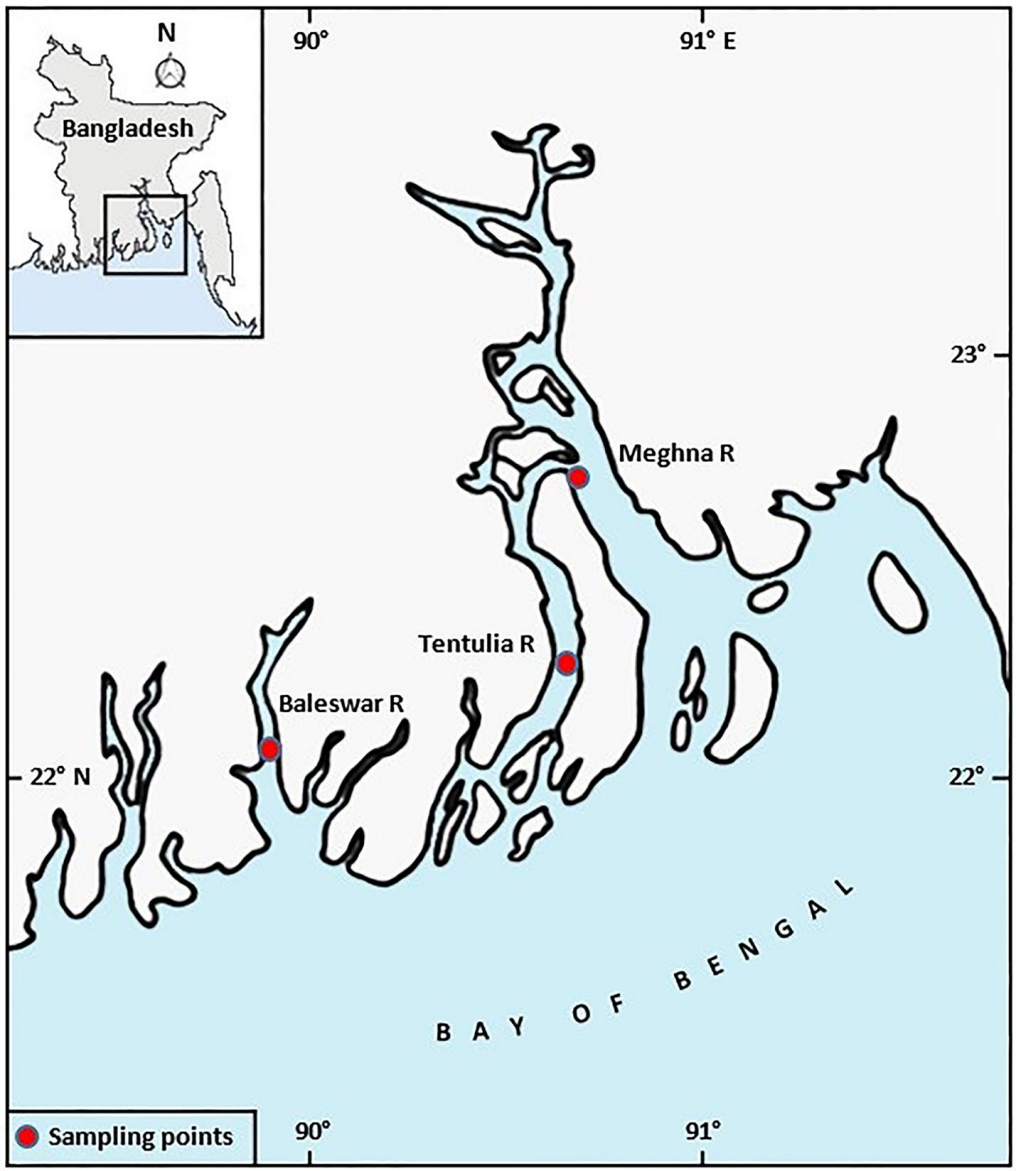
Map showing the collection sites of *S. panijus* from the rivers Baleswar, Tentulia and Meghna in southwest coast of Bangladesh

Altogether 194 specimens of *S. panijus* were collected from three rivers comprising 71 individuals from Meghna river (Daulatkhan, 22°73′N and 89°68E), 61 individuals from Tentulia river (Burhanuddin, 22°33′N and 90°65′E) and 62 individuals from Baleswar river (Pirojpur, 22°13′N and 89°90′E) by using trammel net and trawl net from January to March, 2015 (Fig. 1). Immediately after collection, samples were transported in ice cooled boxes to the laboratory at Patuakhali Science and Technology University for identification and morphometric measurements. Identification of fish was based on phenotypic characteristics. The sex was identified by visual inspection of gonads and external sexual characteristics.

### Morphometric data

**Fig. 2:**
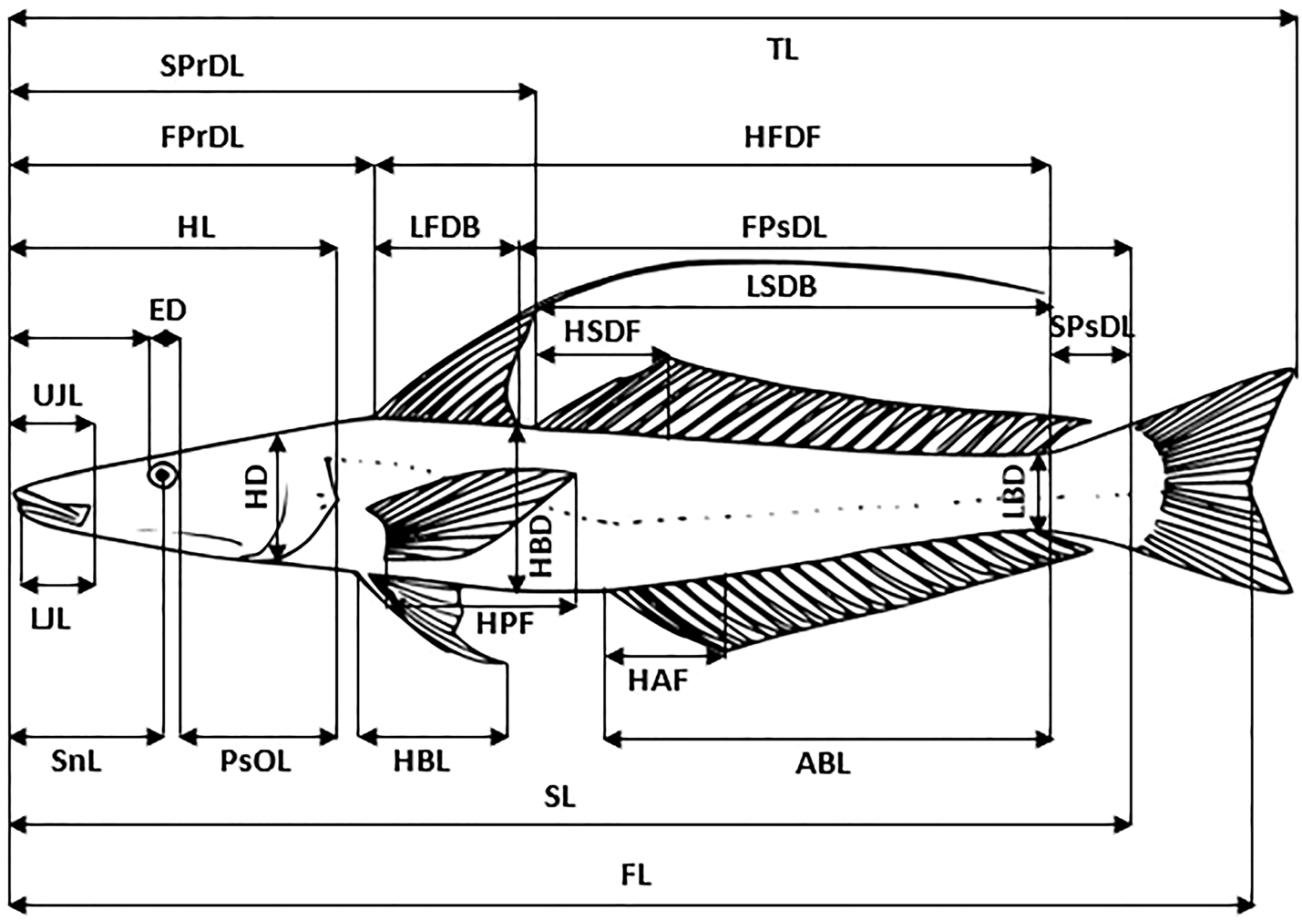
Overview of different morphometrics investigated in *S. panijus*

A total of 27 morphometric characters were measured using digital callipers (Fig. 2). The measured characters are: total length (TL), fork length (FL), standard length (SL), head length (HL), head depth (HD), highest body depth (HBD), lowest body depth (LBD), pre-orbital length (PrOL), post-orbital length (PsOL), eye diameter (ED), snout length (SnL), first pre-dorsal length (FPrDL), second pre-dorsal length (SPrDL), first post-dorsal length (FPsDL), second post-dorsal length (SPsDL), height of first dorsal fin (HFDF), height of second dorsal fin (HSDF), height of pectoral fin (HPF), height of ventral fin (HVF), height of anal fin (HAF), length of first dorsal base (LFDB), length of second dorsal base (LSDB), length of pectoral base (LPB), length of ventral base (LVB), length of anal base (LAB), upper jaw length (UJL) and lower jaw length (LJL).

### Statistical analysis

As variation should be attributable to body shape differences, and not correlated to the relative size of the fish, an allometric method suggested by Elliott et al. (1995) was used to correct size-dependent variation in morphometric characters:

$${\text{M}}_{\text{adj}} = {\text{M}}({\text{L}}_{\text{s}} /{\text{L}}_{0} )^{\text{b}}$$

where, M is the original measurement, M_adj_ is the size adjusted measurement, L_0_ is the standard length of the fish, L_s_ is the overall mean of the standard length for all fish from all samples in each analysis, and b is estimated for each character from the observed data as the slope of the regression of log M on log L_0_ using all fish from both groups. The significance of correlation test between transformed variables and total length was used to confirm the result derived from allometric method.

Univariate ANOVA, linear discriminate function analysis (DFA), principal component analysis (PCA) and cluster analysis (CA) were performed to assess the significant variation among the morphometric characters between the populations. Statistical Package for Social Science (SPSS) version 16 software package, STATISTICA and Microsoft excel, 2013 were used for data analysis and graph making.

## Results

The descriptive data of length and weight comprising mean values of minimum and maximum ranges, and standard deviation for each sample are presented in Table 1. Out of 27 morphological characters, 10 characters showed significant difference (*p* < 0.05) among the populations of *S. panijus* of the Meghna, Tentulia and Baleswar rivers (Table 2) and these significant traits were further used for discriminate function analysis (DFA), principal component analysis (PCA) and cluster analysis (CA).

**Table 1:** Descriptive data of the *S. panijus* sampled from the Meghna, Tentulia and Baleswar rivers in the southwest Bangladesh

Sampling site	Sex	Total length (min–max)	Total length (mean ± SD)	Weight (min–max)	Weight (mean ± SD)
Meghna river	Male (42)	14.9–27.6	21.18 ± 3.57	29.47–83.42	52.87 ± 13.10
	Female (29)	15.5–28.7	22.08 ± 3.76	28.96–82.18	46.50 ± 13.55
Tentulia river	Male (33)	15.5–29.3	21.78 ± 3.83	28.28–86.10	49.32 ± 13.36
	Female (28)	14.9–28.8	20.98 ± 3.78	28.20–78.96	45.43 ± 13.81
Baleswar river	Male (34)	15.0–27.2	21.15 ± 3.47	27.83–86.21	50.61 ± 13.28
	Female (28)	15.4–27.7	20.98 ± 3.55	26.33–80.02	47.79 ± 13.09

**Table 2:** Descriptive statistics of univariate ANOVA based on morphometric characters of *S. panijus*

Morphometric characters	Wilks lambda	*F* value	*p* value	Morphometric characters	Wilks lambda	*F* value	*p* value
TL	1.000	0.039	0.961	SPsDL	0.997	0.265	0.767
FL	1.000	0.021	0.979	HFDF	0.999	0.105	0.901
SL	1.000	0.045	0.956	HSDF	1.000	0.024	0.976
HL	0.994	0.598	0.551	HPF	0.995	0.490	0.614
HD	0.920	8.310	0.000*	HVF	0.993	0.710	0.493
HBD	0.953	4.673	0.010*	HAF	0.916	8.715	0.000*
LBD	0.895	11.123	0.000*	LFDB	0.991	0.873	0.419
PrOL	0.999	0.061	0.941	LSDB	0.967	3.225	0.042*
PsOL	0.943	5.749	0.004*	LPB	0.964	3.531	0.031*
ED	0.865	14.869	0.000*	LVB	0.998	0.233	0.792
SnL	0.951	4.863	0.009*	LAB	0.995	0.523	0.593
FPrDL	0.997	0.282	0.755	UJL	0.999	0.106	0.900
SPrDL	0.942	5.806	0.004*	LJL	0.998	0.145	0.865
FPsDL	0.991	0.823	0.441				

* Significant value (*p* < 0.05)

A number of scientists recommended that for PCA and DFA analysis, the ratio of the number of organisms measured (N) relative to the parameters included (P) should be at least 3–3.5 (Kocovsky et al. 2009). In this study, for multivariate analysis, we used only morphometric characters that were significant at a high level (*p* < 0.05), and under these circumstances, the N:P ratio was 19.4 (194/10) for these traits including HD, HBD, LBD, PsOL, ED, SnL, SPrDL, HAF, LSDB and LPB. Appropriateness of the data were examined for PCA through Bartlett’s test of sphericity and it was significant (*p* < 0.01). The involvement of variable to principal component were analyzed for examining which morphometric traits creates maximum difference among the populations. In principal component analysis, ten morphometric measurements removed four factors in each sex with eigenvalues > 1, explaining 80.75 and 78.61 % of the variance in male and female respectively (Table 3).

**Table 3:** Eigen values, percentage of variance and percentage of cumulative variance for principal components analysis in the *S. panijus*

Factor	Eigen-values		% of variance		Cumulative variance %	
	Male	Female	Male	Female	Male	Female
PC1	18.168	16.596	67.290	61.466	67.290	61.466
PC2	1.335	2.072	4.944	7.674	72.234	69.139
PC3	1.201	1.491	4.449	5.521	76.684	74.660
PC4	1.098	1.068	4.065	3.954	80.749	78.614

The first principal component (PC1) described 67.29 and 61.47 % of the variation in males and females and the second principal component (PC2) for 4.94 and 7.674 % in males and females, respectively (Table 3). The most significant loadings on PC I were from HD, HBD, LBD, PsOL, SnL, SPrDL, HAF, LSDB, LPB for male and HD, HBD, PsOL, SnL, SPrDL, HAF, LSDB, LPB and on PC II, no trait was significant in male while in female ED and SPrDL were significant (Table 4). The morphometric characters with an eigenvalue above 1 were included and others excluded in this analysis. It is worth mentioning that a factor loading more than 0.30 is considered significant, 0.40 is considered more significant, and factor loadings 0.50 or above is considered very significant (Lombarte et al. 2012). In our present study, significant factors considered only those factors with loadings greater than 0.4.

**Table 4:** Factor loadings of principal components based on morphometric characters in the *S. panijus* from southwest Bangladesh

Characters	PC I		PC II		PCIII		PCIV	
	Male	Female	Male	Female	Male	Female	Male	Female
HD	0.765	0.738	–	–	–	–	–	–
HBD	0.837	0.679	–	–	–	–	–	–
LBD	0.732	–	–	–	–	–	–	0.449
PsOL	0.714	0.913	–	–	−0.575	–	–	–
ED	–	–	–	0.837	–	–	0.798	–0.436
SnL	0.854	0.861	–	–	–	–	–	–
SPrDL	0.945	0.767	–	0.412	–	–	–	–
HAF	0.692	0.665	–	–	0.403	–	–	–
LSDB	0.872	0.953	–	–	–	–	–	–
LPB	0.644	0.737	–	–	0.453	–	–	–

**Fig. 3:**
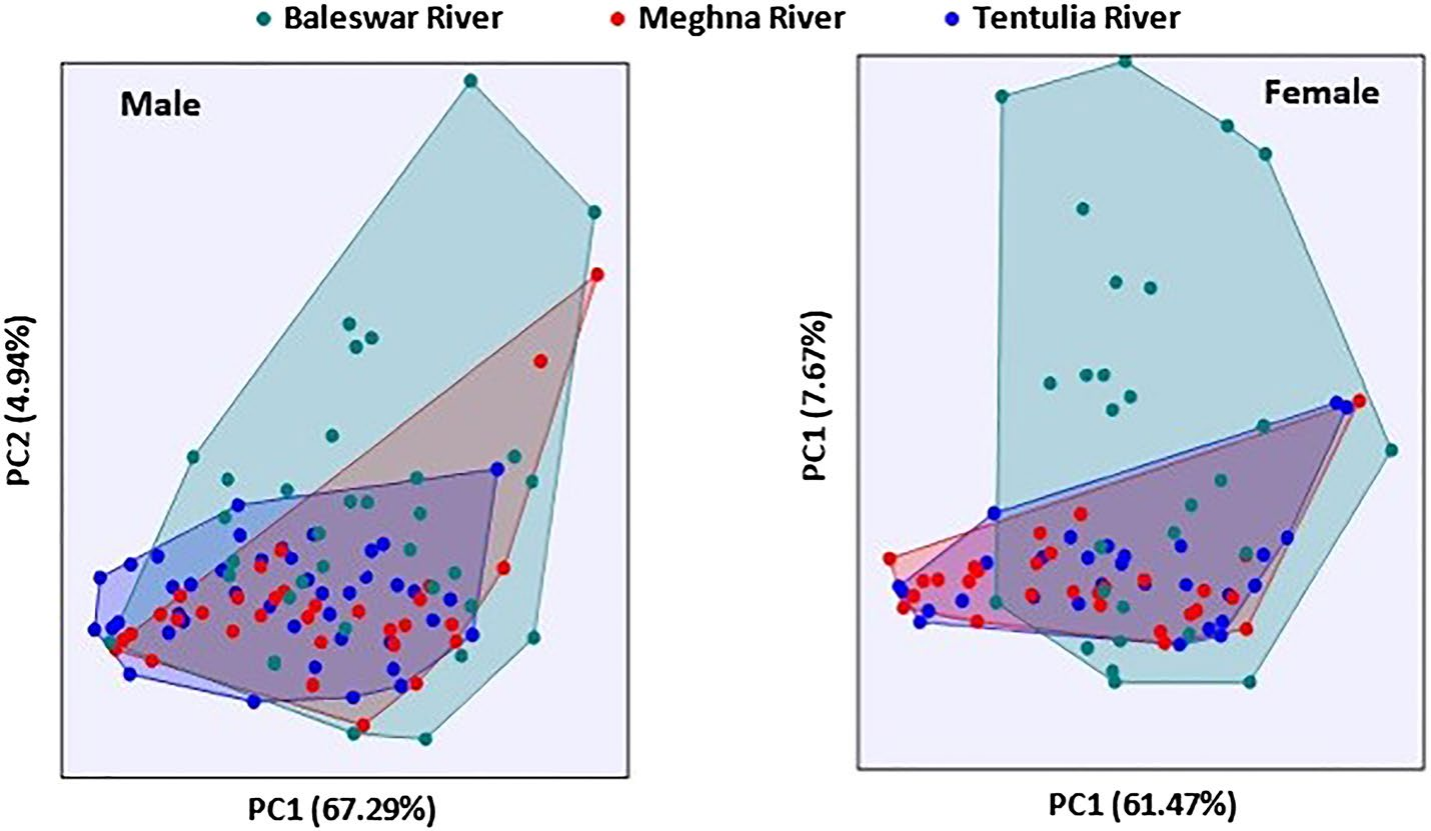
Scatterplot of the scores from PC1 and PC2 for morphometric characters of male and female *S. panijus* collected from the river Baleswar, Meghna and Tentulia in southwest coast of Bangladesh

Visual investigation of plotted of PC1 and PC2 scores showed that the male samples were grouped into three areas but with high degree overlap among the three rivers. But in female visual examination of plots of PC1 and PC2 scores, specimens were grouped into three areas with highly overlap between the Meghna and the Tentulia River but less overlapped with the Baleswar river stations (Fig. 3).

In discriminant function analysis, Wilks’ lambda test showed significant differences in 10 characters out of 27 morphometric characters of the three populations in both sexes. In this test, among the three stations one function of each sex was highly significant (*p* < 0.01) (Table 5).

**Table 5:** Wilks’ lambda test for verifying differences between sexes of *S. panijus* with morphometric measurements using DFA

Group	Functions	Wilks’ lambda	Chi square	df	*p*
Male	1 through 2	0.418	88.519	20	0.000
	2	0.832	18.721	9	0.028
Female	1 through 2	0.343	82.887	20	0.000
	2	0.948	4.160	9	0.901

DFA revealed that 71 of 109 male (71.1 %) and 60 of 85 female (70.6 %) were original correctly classified in their respective grouped whereas 68.8 % of male and 56.5 % of female cross validated group were correctly classified. Medium classification success rates were obtained from male for the Meghna estuary (73.8 %), Tentulia estuary (66.7 %) and Baleswar estuary (64.7 %) while female for the Meghna estuary (48.3 %), Tentulia estuary (53.6 %) and Baleswar estuary (67.9 %) stocks representing a medium correct classification of individuals into their original populations regarding morphometric characters (Table 6).

**Table 6:** Classification of individuals into their original population using classification matrix of the DFA based on morphometric measurements

	Meghna		Tentulia		Baleswar		Total	
	Male	Female	Male	Female	Male	Female	Male	Female
*Original group*								
Meghna river	34	17	8	11	0	1	42	29
%	81.0	58.6	19.0	37.9	0	3.4	100	100
Tentulia river	6	10	26	18	1	0	33	28
%	18.2	35.7	78.8	64.3	3	0	100	100
Baleswar river	7	1	3	2	24	25	34	28
%	20.6	3.6	8.8	7.1	70.6	89.3	100	100
*Cross validated*								
Meghna river	31	14	10	14	1	1	42	29
%	73.8	48.3	23.3	48.3	2.4	3.4	100	100
Tentulia river	10	11	22	15	1	2	33	28
%	30.3	39.3	66.7	53.6	3.0	7.1	100	100
Baleswar river	8	7	4	2	22	19	34	28
%	23.5	25.0	11.8	7.1	64.7	67.9	100	100

**Fig. 4:**
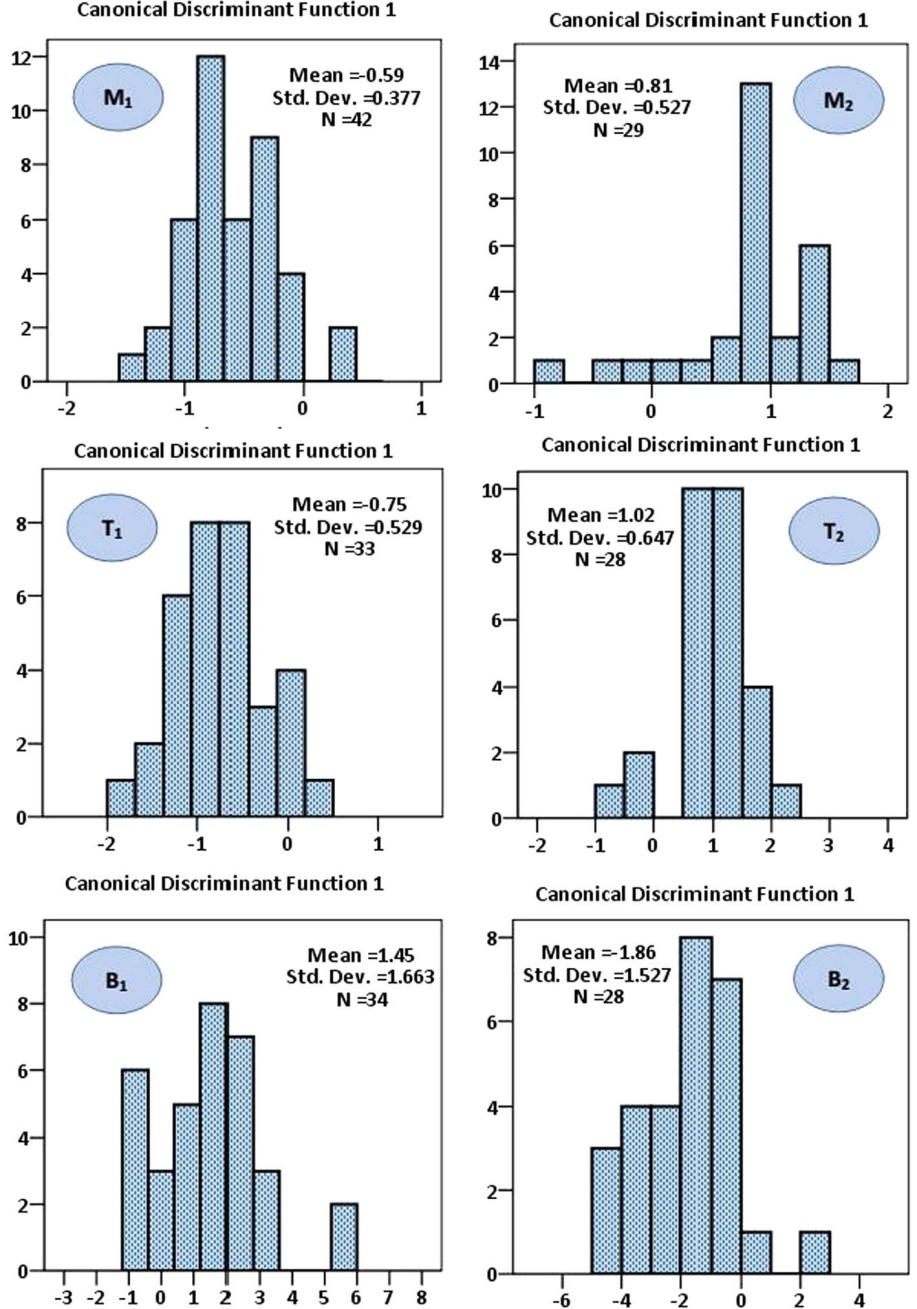
Coordinate plot for the three studied population of *S. panijus* according to the first two discriminant functions. **M**_**1**_ Meghna-male, **M**_**2**_ Meghna-female; **T**_**1**_ Tentulia-male, **T**_**2**_ Tentulia-female and **B**_**1**_ Baleswar-male, **B**_**2**_ Baleswar-female

There was a high degree of separation and some ranges of overlap among the three populations and between the sexes of *S. panijus* population in the study (Fig. 4). Morphometric traits that demonstrated significant differences in multivariate analysis for male and female populations were considered for the stock delineation.

**Fig. 5:**
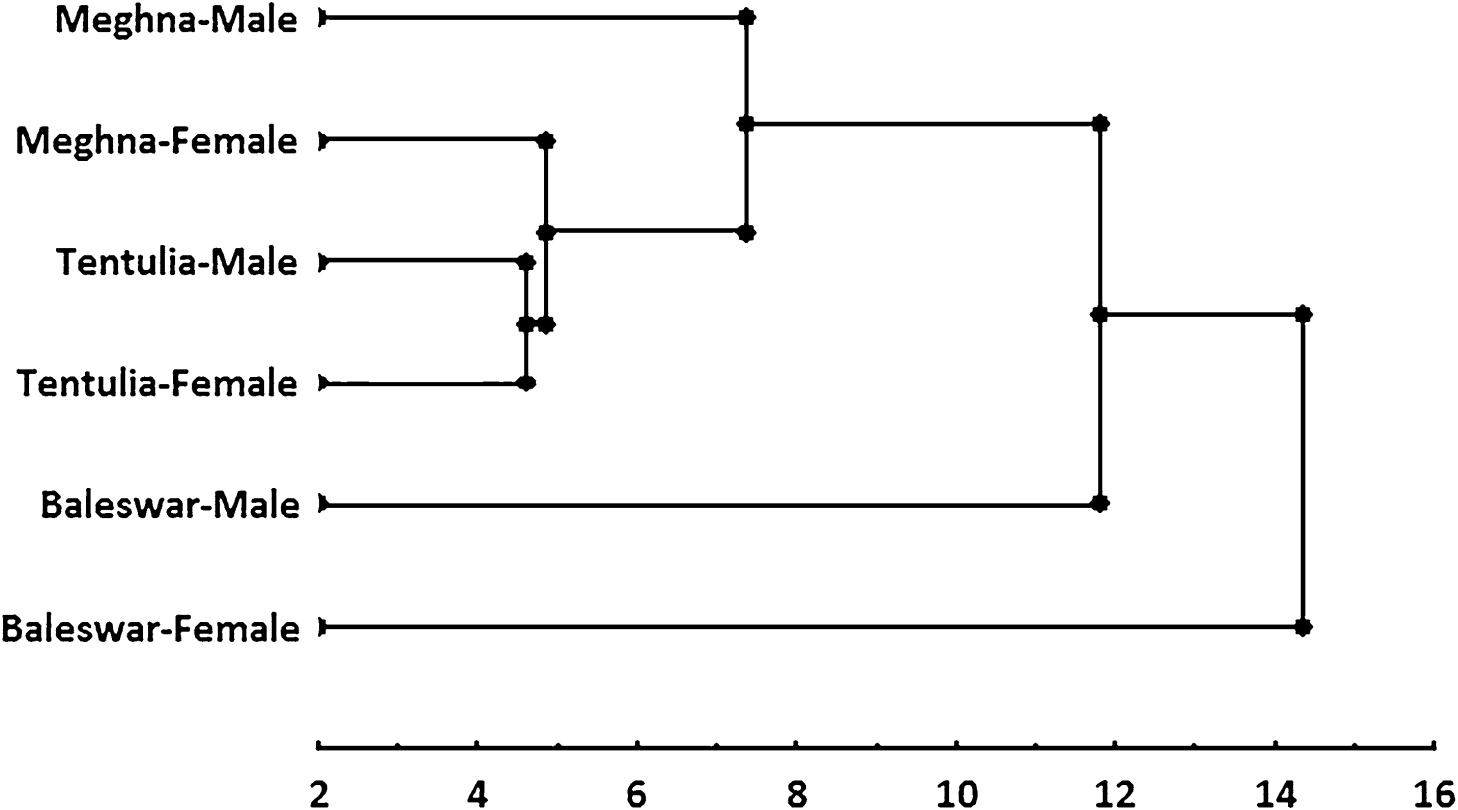
Dendrogram derived from cluster analyses of morphometric measurements for different sexes of *S. panijus* from the river Baleswar, Meghna and Tentulia in southwest coast of Bangladesh

The dendrogram drawing based on euclidean distances between the groups of centroids using an UPGMA displayed two main clusters: Meghna (male), Meghna (female), and Tentulia (male) and Tentulia (female) in one group and Baleswar (male) and Baleswar (female) in the other group. Also, the results of this analysis demonstrated Meghna (male and female) and Tentulia (male and female) closed together and far from Baleswar (male and male), although they are separated geographically (Fig. 5). However, Meghna and Tentulia are closer together and are not highly geographically separated in comparison with Baleswar.

## Discussion

The results obtained from the present investigation demonstrated that there are significant phenotypic variations among the three studied populations as well as between the sexes. Analysis of univariate ANOVA showed that 10 out of 27 transformed morphometric characters of *S. panijus* were significantly different with each of the three river populations. The actual reasons for morphometric variation between populations are often fairly tough to describe. However, several studies have found variations because of the environmental conditions, geographic position, ecological change and sometimes human error during work on morphometric measurements and meristic counts (Cabral et al. 2003; Díaz de Astarloa et al. 2011; Chaklader et al. 2016a).

Discriminant function analysis (DFA) could be a suitable method to differentiate different stocks of the same species, which could be of concern to stock management programs (Karakousis et al. 1991). In the present study, a high classification of individuals which were classified correctly into their own groups by DFA was accomplished (Fig. 4), and this separation was somewhat confirmed by PCA. Though, there were some arrays of overlap somewhat found in all of the characters which was examined between three groups. Vatandoust, et al. (2015) compared the morphometric characteristics between two groups of Caspian lamprey, *Caspiomyzon wagneri* (Pisces: Petromyzontidae), and noted some ranges of overlap in multivariate analysis. It is well known that phenotypic traits can demonstrate high plasticity due to changes in environmental conditions (Swain and Foote 1999). This study showed that the population differentiation which resulted from several multivariate analyses in females was higher than that of males. Similar results was perceived during the study on morphometric dissimilarity of Caspian lamprey from the migrating stocks of two rivers along the southern Caspian Sea (Vatandoust et al. 2015).

This discrimination was ensured by another multivariate analysis PCA, where pictorial analysis of plotted PC1 and PC2 scores for every specimen showed that three samples had a comparatively high degree of intersection among the three locations in male with a high degree of intersection between the Meghna and the Tentulia River but less overlap with the Baleswar River with respect to morphometric characters. Fish samples from the Meghna and the Tentulia River population were morphometrically similar suggesting that may be due to the movement and mixing of both populations. The population of Baleshwar River was very different from the other populations which was possibly due to the large distances from the remaining two Rivers. Moreover, the Baleswar river population was geographically isolated from others which could have thwarted the movement of fish from intermingling with populations in other rivers. This inter-population variation may be attributed due to distance between the rivers, separate geographical location as well as the environmental constrains experienced by each population. Konana et al. (2010) applied PCA on the populations of freshwater shrimp *Macrobrachium vollenhovenii* collecting from Côte d’Ivoire Rivers and reported notable morphometric variation due to distance and geographical location of rivers. Paugy and Lévêque (1999) also showed that populations of same species originating from different geographical areas were morphologically different.

The variation among the stocks of three river populations could be a consequence of phenotypic plasticity in response to unusual hydrological conditions such as differences in alkalinity, current pattern, temperatures, turbidity, and salinity, as well as the intermingling relationship between stocks that may be due to their homogenous habitat attributes and to environmental impacts. A similar study was conducted by Mir et al. (2013) who reported variation among the *Labeo rohita* stocks of Gangabasin due to uncommon hydrological conditions such as differences in alkalinity, current pattern, temperatures, turbidity and the closeness among the stocks due to their similar habitat attributes and to environmental impacts. The environmental parameters, especially salinity, in Tentulia and Meghna rivers were almost the same in comparison with Baleswar river. Dasgupta et al. (2014) reported that the salinity of Tentulia and Meghna river were 3.5 ppt and 6 ppt respectively while it was 0.6 ppt in Baleswar river which might be the possible cause for variation. Ferrito et al. (2007) stated that morphological discrimination in various populations were highly influenced by habitat differences.

It has been recommended that genetic and environmental factors, as well as their interaction influence the morphological characteristics of fishes (Poulet et al. 2004). The effect of environmental factors, including temperature, salinity, migration distance, and availability of food in their territory, can potentially determine morphometric discrimination of fishes (Turan et al. 2006). The significance of these factors on morphological differentiation in fish species is well known (Akbarzadeh et al. 2009) from previous different study. Generally environmental factors remain predominant during the early life stage of organisms, when the individual’s phenotype is more sensitive to environmental influence and this is of particular importance (Pinheiro et al. 2005). The variability of phenotype may not essentially reflect on the population differentiation at the molecular level (Ihssen et al. 1981). Outwardly the river impoundment fragmentation can lead to an augmentation of pre-existing genetic differences which provide a high inter-population structuring (Esguicero and Arcifa 2010). Hence, there is the probability that the observed morphological divergence among the populations in the present study might be due to genetically differences.

During discriminant analysis, an average of 70.9 % of the original grouped cases were correctly classified and 62.7 % of cross-validations were correctly assigned. In a related study, Turan et al. (2005) was able to correctly classify overall 78 % of six populations of *Clarias gariepinus*. Similarly, Pollar et al. (2007) observed that 95.6 % of original group were correctly classified during the discriminant analysis of *Tor tambroides*, while the cross-validation correctly assigned 93.1 % of the fishes into determined populations.

UPGMA drawing based on euclidian distance coefficient for morphometric traits indicated that populations of *S. panijus* in three rivers fragmented from each other. Hence results of DFA, PCA and dendrogram of UPGMA indicated three phenotypically fragmented in the Meghna, the Tentulia and the Baleswar river populations of *S. panijus* in the coastal area of Bangladesh. It seems that isolation by distance appears to be the mechanism liable for population differentiation of *S. panijus*. When a species has a more or less continuous distribution across a range, the balance between gene flow and the forces which is responsible for population differentiation, for example genetic drift or differential selection, may result in clines, whereby genetic differentiation increases with geographic distance (Pinheiro et al. 2005).

## Conclusions

Since the identification of populations and their connectivity between each other is a major point for sustainable management and conservation of species, the use of morphological characters as baseline information appears promising in this region. The present study affords elementary information about the variation of *S. panijus* populations in the coastal rivers of Bangladesh and it recommends that use of morphometric characters generate reliable information for stock discrimination of *S. panijus*, and fish collected from different sites in the present study belonged to different stocks. The findings of the study would serve as primary information of the stock management and enable efficient management strategies for the distinct stocks of *S. panijus* populations in order to make its fishery sustainable and develop appropriate conservation plans in near future.
